# Production of Mucosally Transmissible SHIV Challenge Stocks from HIV-1 Circulating Recombinant Form 01_AE *env* Sequences

**DOI:** 10.1371/journal.ppat.1005431

**Published:** 2016-02-05

**Authors:** Lawrence J. Tartaglia, Hui-Wen Chang, Benjamin C. Lee, Peter Abbink, David Ng’ang’a, Michael Boyd, Christy L. Lavine, So-Yon Lim, Srisowmya Sanisetty, James B. Whitney, Michael S. Seaman, Morgane Rolland, Sodsai Tovanabutra, Jintanat Ananworanich, Merlin L. Robb, Jerome H. Kim, Nelson L. Michael, Dan H. Barouch

**Affiliations:** 1 Center for Virology and Vaccine Research, Beth Israel Deaconess Medical Center, Boston, Massachusetts, United States of America; 2 Ragon Institute of MGH, MIT, and Harvard, Cambridge, Massachusetts, United States of America; 3 U.S. Government Military HIV Research Program (MHRP), Walter Reed Army Institute of Research, Silver Spring, Maryland, United States of America; 4 International Vaccine Institute, Seoul, Korea; Emory University, UNITED STATES

## Abstract

Simian-human immunodeficiency virus (SHIV) challenge stocks are critical for preclinical testing of vaccines, antibodies, and other interventions aimed to prevent HIV-1. A major unmet need for the field has been the lack of a SHIV challenge stock expressing circulating recombinant form 01_AE (CRF01_AE) *env* sequences. We therefore sought to develop mucosally transmissible SHIV challenge stocks containing HIV-1 CRF01_AE *env* derived from acutely HIV-1 infected individuals from Thailand. SHIV-AE6, SHIV-AE6RM, and SHIV-AE16 contained *env* sequences that were >99% identical to the original HIV-1 isolate and did not require *in vivo* passaging. These viruses exhibited CCR5 tropism and displayed a tier 2 neutralization phenotype. These challenge stocks efficiently infected rhesus monkeys by the intrarectal route, replicated to high levels during acute infection, and established chronic viremia in a subset of animals. SHIV-AE16 was titrated for use in single, high dose as well as repetitive, low dose intrarectal challenge studies. These SHIV challenge stocks should facilitate the preclinical evaluation of vaccines, monoclonal antibodies, and other interventions targeted at preventing HIV-1 CRF01_AE infection.

## Author Summary

In this study, we generated and evaluated novel simian-human immunodeficiency virus (SHIV) challenge stocks expressing *env* sequences from HIV-1 strains from Thailand. The lack of such SHIV challenge stocks has been a major unmet need for the field and has hindered progress to evaluate strategies aimed at preventing HIV-1 infection for this region of the world. The challenge stocks described in this manuscript should prove useful for studying the preclinical efficacy and mechanisms of vaccines, antibodies, and other interventions.

## Introduction

The generation and evaluation of simian-human immunodeficiency virus (SHIV) challenge stocks are important for preclinical testing of vaccines, microbicides, monoclonal antibodies, and other interventions aimed at preventing HIV-1 infection. SHIVs are typically constructed with an SIV backbone and HIV-1 *env*, *tat*, *rev*, and *vpu* [1–3]. The predominant HIV-1 subtype in the United States and Europe is subtype B [4]. In contrast, circulating recombinant form 01_AE (CRF01_AE) viruses are predominant in Southeast Asia, including Thailand [5]. The RV144 study in Thailand has been the only partially successful vaccine clinical efficacy trial to date [6]. A SHIV challenge stock expressing CRF01_AE *env* would therefore be critical for testing concepts related to the protection observed in RV144. Studies involving correlates of risk and molecular sieve analyses have raised important hypotheses for how the RV144 vaccine might have worked [7,8], but mechanistic studies have been limited by the lack of an available CRF01_AE SHIV challenge stock. We therefore sought to produce mucosally transmissible SHIVs containing early CRF01_AE *env* sequences.

HIV-1 transmission across a mucosal barrier is a relatively inefficient process and typically results in propagation of only a single transmitted/founder (T/F) virus [9]. A single T/F virus has been deemed responsible for propagation of HIV-1 in 60–80% of mucosal infections [10,11]. SHIVs encoding early or acute HIV-1 *envs* might therefore optimally model acute infection and thus be relevant for testing vaccines and other interventions aimed at preventing mucosal infection.

Three challenge stocks, SHIV-AE6, SHIV-AE6RM, and SHIV-AE16, were generated in human or rhesus PBMC without *in vivo* passaging. These viruses displayed CCR5 tropism and a neutralization phenotype consistent with tier 2 primary isolates. They established mucosal infection in rhesus monkeys, exhibited robust acute viral replication, and maintained chronic viremia in a subset of animals challenged by the intrarectal (i.r.) route. These novel CRF01_AE SHIV challenge stocks should facilitate preclinical mechanistic and efficacy studies involving interventions aimed at preventing infection with CRF01_AE HIV-1.

## Materials and Methods

### Animals

Indian origin rhesus monkeys (*Macaca mulatta*) were used in the current study. Eight Mamu-A*01-negative adult male and female animals were housed at Bioqual (Rockville, MD), and 16 Mamu-A*01-positive adult male and female animals were housed at the New England Primate Research Center (Southborough, MA).

### Ethics

All animals were maintained in accordance with the Association for Assessment and Accreditation of Laboratory Animals with approvals from the relevant Institutional Animal Care and Use Committees (IACUC). The Harvard Medical School Standing Committee on Animals (#03970) and the Bioqual IACUC (14-3131-62P) approved these studies. The animal protocols adhered to NIH standards set forth in the "Policy on Humane Care of Vertebrate Animals Used in Testing, Research, and Training" and the "Guidelines for the Care and Use of Laboratory Animals" (DHHS publication #NIH 85–23). Animal welfare was maintained by the NEPRC and Bioqual animal management programs, which are accredited by the American Association for the Accreditation of Laboratory Animal Care (AAALAC) and meet all applicable federal and institutional standards for standard housing, standard monkey diet, water ad libitum, social enrichments, and steps intended to minimize suffering, such as the use of anesthesia for all procedures and analgesia for invasive procedures such as biopsies. Animals were euthanized by ketamine followed by sodium pentobarbital, which is consistent with the recommendations of the Panel on Euthanasia of the American Veterinary Medical Association.

### Cells

Human and rhesus monkey peripheral blood mononuclear cells (PBMC) were isolated by Ficoll-Hypaque gradient purification, followed by stimulation with concanavalin A (Con A; 6.25 μg/ml) and human interleukin-2 (20 units/ml IL-2; AIDS Research and Reference Reagent Program) overnight. Human PBMCs were purchased commercially (Research Blood Components). Cells were cultured in RPMI 1640 medium supplemented with 20% fetal bovine serum, 2 mM glutamine, 100 units/ml penicillin, 100 μg/ml streptomycin, and 20 units/ml of IL-2. TZM-bl cells (JC53-bl clone 13 cells) are derived from a HeLa cell line (JC.53) that stably expressed CD4 and HIV coreceptors, as well as luciferase and β-galactosidase under the control of the HIV-1 long terminal repeat. TZM-bl cells and GHOST cells expressed CD4 alone or with different chemokine receptors and were obtained from the NIH AIDS Research and Reference Reagent Program.

Lymph node and colorectal biopsies were collected at week 12 and incubated in RPMI 1640 containing 10% FBS supplemented with 200 U/ml of type IV collagenase (Sigma-Aldrich) and 30 U/ml of DNase I (Sigma-Aldrich) at 37°C with rocking for 30 min. The digested biopsy tissues were then homogenized, and the solution was strained with a 70-μm (pore size) cell strainer (BD Falcon). The cell suspension was then centrifuged at 695 × *g* for 25 min on a 35% Percoll (Sigma-Aldrich) gradient. Pellets containing lymphocytes were collected and processed for assays. Proviral DNA assays were conducted using the QIAcube HT (Qiagen) performed as previously described [12].

### Construction of SHIV molecular clones

Twenty-nine HIV-1 CRF01_AE early *env* sequences (AE1 –AE29) derived from acutely HIV-1-infected participants in either the RV217 Early Capture HIV Cohort Study in East Africa and Thailand (Merlin Robb, AIDS Vaccine 2012) or the RV254/SEARCH study in Thailand [13] were used to generate SHIV molecular clones (Table 1). The *env* sequences were synthesized by GeneArt (GeneArt, Invitrogen) and inserted into the KB9-AC plasmid as described [14]. Plasmids were sequenced and transfected into 293T cells using LipoD293 (SignaGen Laboratories). Cell culture supernatants were collected after 48 h and clarified through a 0.45 μm filter.

**Table 1:** HIV-1 ENV sequence characteristics.

Env	Sequence ID	Genbank	Study	Subtype	Viral Load	Fiebig
AE1	40363v03_01	KU230424	RV217	AE	5.89 X 10^5^	I-II
AE2	254022_P00c	KU230435	RV254	AE	3.35 X 10^5^	V
AE3	254034_P00h	KT185829	RV254	AE	4.11 X 10^6^	III
AE4	40250v03_02R	KU230422	RV217	AE	2.40 X 10^5^	I-II
AE5	254016_P00a	KT185701	RV254	AE	3.88 X 10^5^	III
AE6	40100v01_06	KU230419	RV217	AE	8.91 X 10^5^	I-II
AE7	40061v03_06	KU230417	RV217	AE	1.82 X 10^4^	I-II
AE8	40436v02_01	KU230426	RV217	AE	2.09 X 10^5^	I-II
AE9	254020_P00Ra	KU230433	RV254	AE	7.65 X 10^5^	III
AE10	254019_P00a	KT185951	RV254	AE	3.67 X 10^7^	II
AE11	40094v01_01R	KU230418	RV217	AE	1.32 X 10^4^	I-II
AE12	40123v03_06R	KU230420	RV217	AE	5.13 X 10^4^	I-II
AE13	254021_P00c	KU230434	RV254	AE	5.11 X 10^5^	V
AE14	40363v03_09	KU230425	RV217	AE	5.89 X 10^5^	I-II
AE15	254014_P00Rb	KU230430	RV254	AE	2.32 X 10^3^	I
AE16	254004_P00Rc	KT185994	RV254	AE	5.71 X 10^5^	IV
AE17	40231v02_02R	KU230421	RV217	AE	6.31 X 10^5^	I-II
AE18	254006_P00Ra	KU230428	RV254	AE	5.52 X 10^6^	III
AE19	254018_P00a	KU230432	RV254	AE	1.26 X 10^5^	II
AE20	254007_P00Ra	KU230429	RV254	AE	4.82 X 10^5^	I
AE21	254002_P00Rd	KT185770	RV254	AE	3.19 X 10^6^	II
AE22	40353v04_01R	KU230423	RV217	B	2.40 X 10^4^	I-II
AE23	254001_P00Ra	KU230427	RV254	AE	7.35 X 10^6^	III
AE24	254014_P00Rh	KU230431	RV254	AE	2.32 X 10^3^	I
AE25	254023_P00a	KT185973	RV254	AE	3.08 X 10^7^	III
AE26	254026_P00Ra	KU230436	RV254	AE	5.47 X 10^3^	I
AE27	254027_P00Ra	KU230437	RV254	AE	2.54 X 10^4^	I
AE28	254029_P00b	KT186030	RV254	AE	1.18 X 10^5^	II
AE29	254036_P00a	KT185894	RV254	AE	2.33 X 10^7^	IV

### Generation of large-scale SHIV stocks

Eight infectious molecular clones (AE3, AE4, AE6, AE15, AE16, AE17, AE21, AE22) that replicated to high levels in human PBMC were selected to generate large-scale challenge stocks. Each virus stock was produced from PBMC isolated from 120 ml of human or rhesus monkey whole blood. Cell culture supernatants harvested from transiently transfected 293T cells were used to inoculate ConA-stimulated PBMC in the presence of 20 U/ml human IL-2 (AIDS Research and Reference Reagent Program). SHIV-AE6RM was prepared by propagation of the human SHIV-AE6 stock in rhesus monkey PBMC. The media was replaced and collected in 3-day increments over the course of 12–15 days. Virus was quantified by SIV p27 enzyme-linked immunosorbent assay (ELISA; Zeptometrix), and the 50% tissue culture infectious does (TCID_50_) was determined in TZM-bl cells.

### TCID50 titer in TZM-bl cells

Virus stocks were assessed for infectivity by inoculation of TZM-bl cells using 5-fold serial dilutions in the presence of 11 μg/ml of DEAE-dextran hydrochloride (Sigma, St. Louis, MO) in quadruplicate wells. Virus infectivity was determined 48 h post infection by measuring the level of luciferase activity expressed in infected cells. The TCID_50_ was calculated as the dilution point at which 50% of the cultures were infected.

### Coreceptor studies

The inhibitors TAK-779 and AMD-3100 were obtained from the AIDS Research and Reference Reagent Program and were diluted per manufacturer instructions. TZM-bl cells were seeded at 3 X 10^4^ cells/well in flat-bottom plates. The cells were incubated for 1 h in 10-fold dilutions of TAK-779 ranging from 5 to 500,000 ng/ml or 10-fold dilutions of AMD-3100 ranging from 5 to 50,000 ng/ml or mock infected with supernatant from cultured PBMC without viral infection. After 1 h, 100 TCID_50_ of the SHIV stocks were added to the wells, and 48 h later the cells were lysed and analyzed by Steady-Glo (Promega) luciferase assay with luminescence measured on a Victor 3 luminometer (Perkin-Elmer). GHOST cells expressing coreceptors CXCR4, CCR5, and CCR5/CXCR4 and the parental strain were obtained from the AIDS Research and Reference Reagent Program. These were maintained in Dulbecco modified Eagle medium with 10% fetal calf serum and penicillin-streptomycin and supplemented with 500 μg/ml G418, 100 μg/ml hygromycin, and 1 μg/ml puromycin. Puromycin was excluded for culture of the parental cell line. Cells were plated at 10^4^ cells per ml in 96-well round-bottom wells in medium supplemented with 20 μg/ml Polybrene. One hundred TCID_50_ of each virus stock was added to each well, followed by incubation overnight. After being washed with phosphate-buffered saline (PBS) and replacement with fresh culture medium, cell culture supernatants were harvested 4 days after incubation and assessed for SIV p27 ELISA (Zeptometrix).

### Neutralization assay

Neutralizing antibody titers against SHIV isolates were determined using a luciferase-based assay in TZM.bl cells as previously described [15,16]. This assay measures a decrease in luciferase reporter gene expression following single-round viral infection of TZM.bl cells. Briefly, 3-fold serial dilutions mAb reagents were performed in duplicate and incubated with primary SHIV isolates for 1 hour at 37°C. TZM.bl cells were then added in growth media containing DEAE-dextran at a final concentration of 11 μg/ml, and assay plates incubated for 48 hours at 37°C, 5% CO_2_. Luciferase reporter gene expression was measured using Bright-Glo luciferase reagent (Promega) and a Victor 3 luminometer (Perkin Elmer). Neutralization titers (50% inhibitory concentrations, IC50) were calculated as the sample dilution at which the relative luciferase units (RLU) were reduced by 50% compared to RLU in virus control wells after subtraction of background RLU in cell control wells. MAbs 4E10, 2G12, 2F5, b12, PG9, PG16, and VRC01 (Polymun Scientific) and soluble human CD4 protein (Progenics) were obtained commercially. MAbs 3BNC117, 8ANC195, 10–1074, 3BC176, and 45-46W were provided by Michel Nussenzweig (The Rockefeller University, New York, NY). MAbs PGT121, PGT126, and PGT128 were provided by Dennis Burton (The Scripps Research Institute, La Jolla, CA).

### Intrarectal inoculation of CRF01_AE SHIVs

Animals received a single intrarectal (i.r.) inoculation with 1 ml of undiluted SHIV-AE6, SHIV-AE6RM, or SHIVAE-16 stocks. For the SHIV-AE16 *in vivo* titration, six i.r. challenges with 1 ml of 1:1 or 1:10 diluted virus stock were performed with two week intervals from week 0 to week 10. All animals were monitored for viral loads and CD4^+^ T cell counts.

### CD4+ T cell counts

EDTA-anticoagulated whole blood, PBMC, and lymph node mononuclear cells (LNMC) were stained using anti-CD3-APC, anti-CD4-PE, and anti-CD8-FITC MAbs and analyzed using a FACSCalibur flow cytometer (BD Biosciences, San Jose, Ca). Cell counts were determined using the BD True Count tubes according to the manufacturer’s instructions (BD Biosciences).

### Measurement of plasma viral RNA levels

Viral RNA was isolated from cell-free plasma using the QIAamp viral RNA isolation kit (Qiagen) and quantified as previously described [4,14,17]. Quantitative PCR and viral load assays were performed as previously described [6,12,14]. All viral loads were run at the end of each study to maintain objectivity and ensure that all animals were handled identically.

### Single-genome amplification (SGA) and sequencing

Viral RNA was extracted using a QIAamp viral RNA minikit (Qiagen). RNA was eluted and immediately subjected to cDNA synthesis. Reverse transcription of RNA to single-stranded cDNA was performed using SuperScript III reverse transcriptase (Invitrogen) as described [9,14].

### Nucleotide sequence accession numbers

The *env* sequences of the amplicons were deposited in GenBank under accession numbers KT581024—KT581117 as detailed in Table 1.

## Results

### Generation of CRF01_AE SHIV stocks

Twenty-nine CRF01_AE early HIV-1 *env* sequences from acutely HIV-1 infected individuals from Thailand (Table 1) were cloned into the KB9-AC backbone, essentially as described [14]. Plasmids were used for transfections in 293T cells to generate virus, and virus infectivity titers were determined (Table 2). Culture supernatants were used to assess viral growth in human and rhesus PBMC. As is the case with other SHIVs [10,11,14], these viruses replicated well in human PBMC but inefficiently in rhesus PBMC (Table 2). Interestingly, SHIV-AE22 was identified as the most infectious virus in this set of SHIVs, but this virus was confirmed to be subtype B rather than CRF01_AE (Table 1).

**Table 2:** Cloning and replication of 293T-derived SHIVs in human and rhesus PBMC^a^.

			Human^b^	Rhesus^c^	PBMC
			293T cells	PBMC	pg/ml
Env	Project ID	Subtype	TICD_50_/ml	ng/ml	
AE1	RV217	AE	3.20 X 10^2^	0	0
AE2	RV254	AE	2.56 X 10^3^	33	0
AE3	RV254	AE	5.12 X 10^3^	6.28 X10^2^	0
AE4	RV217	AE	1.02 X 10^4^	6.69 X10^2^	0
AE5	RV254	AE	20	1.90 X10^2^	0
AE6	RV217	AE	2.05 X 10^4^	3.74 X10^2^	3.25 X 10^2^
AE7	RV217	AE	6.40 X 10^2^	3.24 X10^2^	0
AE8	RV217	AE	3.20 X 10^2^	1.39 X10^2^	0
AE9	RV254	AE	2.56 X 10^3^	1.53 X10^2^	0
AE10	RV254	AE	1.28 X 10^3^	24	0
AE11	RV217	AE	2.56 X 10^3^	32	0
AE12	RV217	AE	5.12 X 10^3^	28	0
AE13	RV254	AE	5.12 X 10^3^	44	0
AE14	RV217	AE	1.02 X 10^4^	46	0
AE15	RV254	AE	2.56 X 10^3^	0	0
AE16	RV254	AE	5.12 X 10^3^	2.72 X10^2^	1.25 X 10^2^
AE17	RV217	AE	1.02 X 10^4^	4.16 X10^2^	0
AE18	RV254	AE	2.56 X 10^3^	1.13 X10^2^	0
AE19	RV254	AE	1.60 X 10^2^	1.75 X10^2^	0
AE20	RV254	AE	0	0	0
AE21	RV254	AE	1.64 X 10^5^	5.15 X10^2^	0
AE22	RV217	B	3.28 X 10^5^	3.32 X10^2^	0
AE23	RV254	AE	0	0	0
AE24	RV254	AE	6.40 X 10^2^	10	0
AE25	RV254	AE	6.40 X 10^2^	33	0
E26	RV254	AE	0	0	0
AE27	RV254	AE	2.05 X 10^4^	39	0
AE28	RV254	AE	40	22	0
AE29	RV254	AE	2.50 X 10^4^	25	0

^*a*^ Supernatants were collected 2 days after plasmid transfection in 293T cells and at 6 to 9 days post inoculation of the stocks into human or rhesus PBMC.

^*b*,*c*^ Supernatants were examined by p27 ELISA.

We selected eight viruses (AE3, AE4, AE6, AE15, AE16, AE17, AE21, AE22) that replicated to high levels in human PBMC for the generation of large-scale challenge stocks by inoculation of the 293T transfection-derived supernatants into primary human PBMC. The large-scale challenge stocks ranged in infectivity from 6.4 X 10^2^ to 7.0 X 10^8^ TCID_50_/ml, and viral loads ranged from 3.2 X 10^8^ to 2.1 X 10^9^ RNA copies/ml (Table 3). The challenge stocks were then assessed for their ability to replicate in rhesus PBMC. Only two stocks, SHIV-AE6 and SHIV-AE16, replicated at detectable levels in rhesus PBMC, and SHIV-AE6 exhibited the highest SIV p27 level as determined by ELISA (Table 3). This stock was therefore chosen to produce a large-scale challenge stock in primary rhesus PBMC, which was designated SHIV-AE6RM. The rhesus-derived SHIV-AE6RM stock replicated 114-fold more efficiently than did the human-derived SHIV-AE6 stock in rhesus PBMC (Table 3).

**Table 3:** Summary of large-scale SHIV stocks^a^.

HIV	PBMC	Infectivity titer	Human p27 PBMC (TCID_50_/ml) (ng/ml)	Viral load (RNA copies/ml) (ng/ml)	Rhesus p27 PBMC	Coreceptor usage
AE3	Human	2 X 10^7^	356.0	1.2 X 10^9^	0	CCR5
AE4	Human	3 X 10^6^	384.0	1.1 X 10^9^	0	CCR5
AE6	Human	6 X 10^5^	147.9	2.0 X 10^9^	0.33	CCR5
AE15	Human	6 X 10^2^	127.8	3.2 X 10^8^	0	CCR5
AE16	Human	1 X 10^5^	37.65	2.1 X 10^9^	0.13	CCR5
AE17	Human	6 X 10^5^	298.0	1.3 X 10^9^	0	CCR5
AE21	Human	4 X 10^8^	353.0	1.3 X 10^9^	0	CCR5
AE22	Human	7 X 10^8^	345.0	1.2 X 10^9^	0	CCR5
AE6RM	Rhesus monkeys	3 X 10^6^	225.2	1.7 X 10^10^	37.65	CCR5

^*a*^ PBMC utilized for stock generation, infectivity in TZM-bl cells (TCID_50_/ml), SIV p27 levels in human PBMC, viral load (RNA copies/ml), and SIV p27 levels after inoculation of rhesus PBMC and coreceptor usage are shown

**Fig 1:**
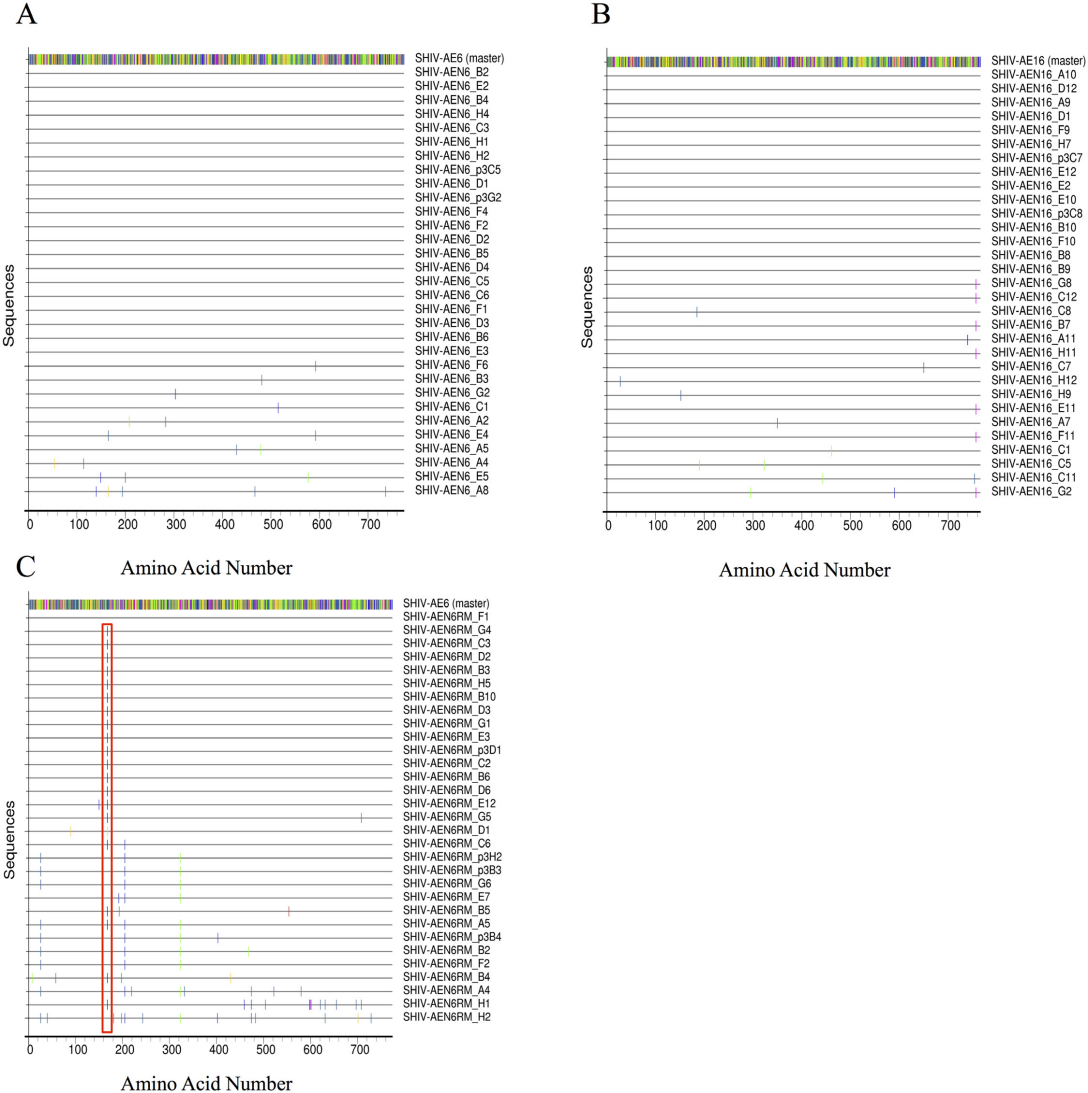
Highlighter amino acid sequence alignment of *env* derived SHIV-AE6, SHIV-AE6RM, and SHIV-AE16 stocks compared to the parental strains. (A) SHIV-AE6, (B) SHIV-AE16, and (C) SHIV-AE6RM amino acid sequences are shown. Amino acids that differ from the parental sequence (GenBank accession numbers KP109513 and KT581086) are indicated in color, while dashes indicate identical amino acid positions. The D168H mutation in SHIV-AE6RM is depicted by the red box. A total of 31 sequences for each SHIV challenge stock was generated by SGA.

We next performed sequencing by single genome amplification (SGA) [11] to analyze the *env* sequences in the SHIV-AE6, SHIV-AE6RM, and SHIV-AE16 challenge stocks as compared to the original HIV-1 *env* sequences from the RV217 and the RV254/SEARCH studies in Thailand. We evaluated 31 SGA-derived sequences obtained for each challenge stock (Fig 1). The SHIV-AE6 stock had one to five random amino acid differences in 10 amplicons (Fig 1A), while the SHIV-AE16 stock had one to three intermittent amino acid differences in 16 amplicons (Fig 1B). The rest of the amplicons for both stocks was identical to the parent sequence. For the rhesus PBMC-derived SHIV-AE6RM challenge stock, 30/31 amplicons contained at least one amino acid difference as compared to the original HIV-1 *env* sequence, and 20/31 amplicons contained a consistent D168H mutation in the V2 loop (Fig 1C). These data show that these SHIV stocks expressed *envs* that were >99% identical to the parental HIV-1 *env* sequences and only contained rare and sporadic amino acid changes, except for the D168H mutation in SHIV-AE6RM.

### Coreceptor usage analysis of the SHIV-AE stocks

**Fig 2:**
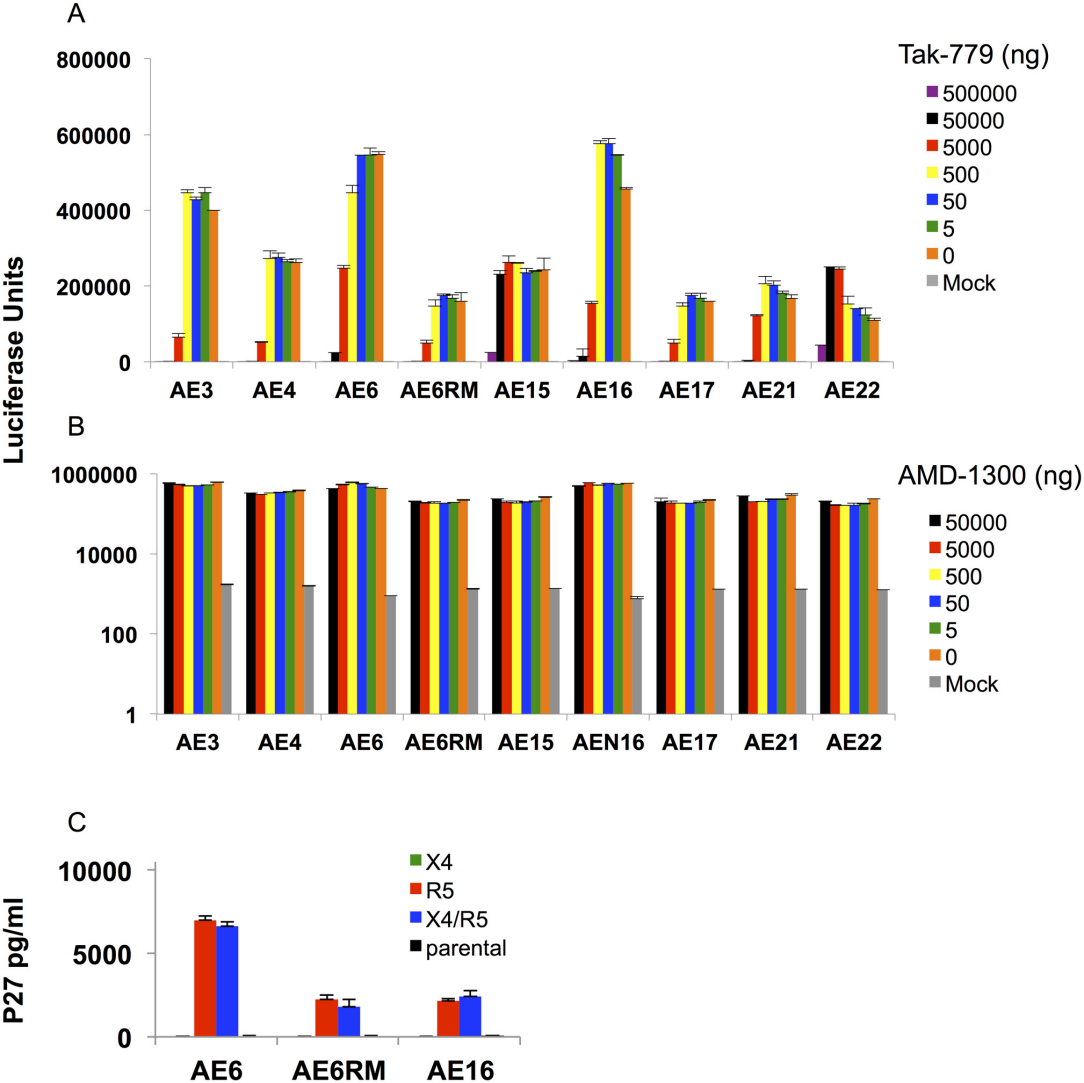
Coreceptor tropism of early SHIV stocks. (A-B) TZM-bl cells were incubated for 1 h with different concentrations of the CCR5 inhibitor TAK-779 or the CXCR4 inhibitor AMD-3100 and subsequently were infected with 100 TCID_50_ of the indicated SHIV stocks. The luciferase activity was quantified after 48 h. (C) GHOST cell lines expressing CXCR4 (X4) and/or CCR5 (R5) coreceptors were used, and inoculated with 100-TCID_50_ SHIV stocks. Cell culture supernatant was collected for SIV p27 determination after 4 days of infection. All assays were done in triplicate. The means with standard deviation are shown.

We examined coreceptor usage of the early CRF01_AE SHIV stocks using TZM-bl cells in the presence of the coreceptor inhibitors TAK779 (CCR5) and AMD3100 (CXCR4). All of the SHIV stocks were potently inhibited by the CCR5 TAK-779 inhibitor (Fig 2A) but were not inhibited by the CXCR4 inhibitor AMD3100 (Fig 2B). We then tested the replication ability of SHIV-AE6, SHIV-AE6RM, and SHIV-AE16 stocks in GHOST cells expressing alternate coreceptors and then measured the SIV p27 production of each SHIV stock in the culture supernatant (Fig 2C). The assay verified that the SHIV stocks replicated in the R5 or dual X4/R5 expressing cells, but did not utilize X4 alone as a coreceptor. The results indicate that these SHIV stocks all utilized the CCR5 coreceptor for cellular entry.

### Antigenic characterization of the SHIV-AE stocks

We next examined the neutralization sensitivity of each SHIV stock in TZM-bl cells with a panel of broadly reactive mAbs (Table 4) targeting the major neutralization epitopes. The SHIV stocks all displayed neutralization profiles consistent with tier 2 neutralization resistant phenotypes, although SHIV-AE6RM showed sensitivity to soluble CD4.

**Table 4:** Neutralization properties of SHIV stocks in TZM-bl assays^a^.

Specificity	Antibody	AE3	AE4	AE6	AE6RM	AE15	AE16	AE17	AE21	AE22
CD4	sCD4	21.52	>50	8.86	0.09	0.80	10.53	18.43	>50	30.84
CD4bs	b12	>50	>50	>25	0.72	>50	>25	>50	>50	>50
	3BNC117	>50	0.55	5.25	1.98	<0.023	>25	1.01	1.87	2.29
	VRC01	>50	4.04	>25	>25	0.04	>25	1.77	3.62	6.88
	45-46W	34.23	1.12	7.75	3.06	NT	6.18	0.86	0.62	1.24
V3/CD4I	3BNC176	>50	>50	>25	>25	NT	>25	>50	>50	>50
GP120 (V3/V4)	2G12	>50	>50	>25	>25	>50	>25	>50	>50	>50
V1/V2 glycan	PG9	3.11	0.64	1.25	7.79	>50	0.03	0.04	<0.02	>50
	PG16	1.92	0.18	0.04	>25	>50	<0.01	0.44	<0.02	>50
V3 glycan	PGT128	43.78	43.51	0.47	1.04	>50	>25	>50	10.09	44.02
	PGT121	>50	>50	13.39	5.75	NT	>25	>50	>50	0.21
	PGT126	>50	>50	0.16	0.16	NT	>25	>50	>50	>50
	10–1074	>50	>50	11.88	2.25	NT	>25	4.21	>50	1.35
gp41/CD4bs	8ANC195	0.57	0.58	0.37	0.41	NT	4.46	0.57	>50	1.10
gp41 (MPER)	2F5	>50	2.83	21.59	>25	2.88	>25	8.10	34.94	24.05
	4E10	24.97	2.76	>25	>25	0.49	15.72	7.29	47.54	>50
	10E8	0.89	0.21	2.95	3.9	NT	6.26	0.36	8.99	5.15
Polyclonal	HIVIG-B	>2,500	1,481.08	2,375.22	2,022.03	296.41	>2,500	>2,500	>2,500	>2,500
	HIVIG-C	587.52	436.21	>625	582.42	191.06	>625	183.14	533.06	601.09

^*a*^CD4bs, CD4 binding site; CD4i, CD4 induced site; HIVIG, purified immunoglobulin obtained from clade B or clade C HIV^+^ plasma samples

### Mucosal infectivity of SHIV-AE6, SHIV-AE6RM, SHIV-AE16 stocks in rhesus monkeys

**Fig 3:**
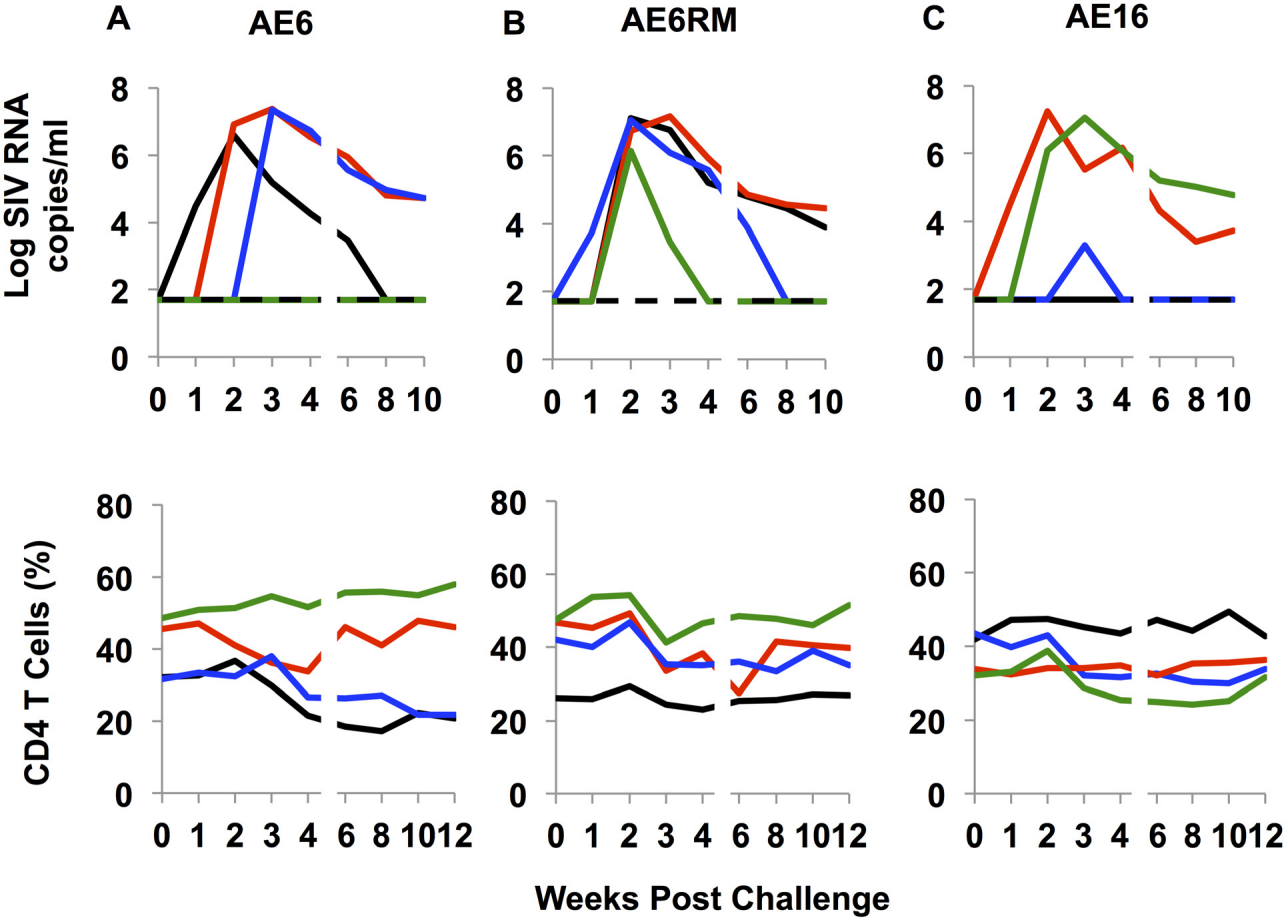
i.r. challenge with SHIV-AE6, SHIV-AE6RM, SHIV-AE16 stocks in rhesus monkeys. Twelve animals were challenged once with 1 ml of undiluted (A) SHIV-AE6 (n = 4), (B) SHIV-AE6RM (n = 4), and (C) SHIV-AE16 (n = 4) stocks by the i.r. route. The upper panel shows plasma viral loads, and the lower panel shows the percentage of CD4^+^ T cells in peripheral blood. The dotted line reflected the limit of detection of the assay (50 RNA copies/ml).

We next assessed the capacity of SHIV-AE6, SHIV-AE6RM, and SHIV-AE16 stocks that displayed detectable levels of replication in rhesus PBMC for their ability to infect rhesus monkeys *in vivo* by the i.r. route. Twelve adult rhesus monkeys were challenged with a single i.r. inoculation of 1 ml of undiluted virus for each stock (n = 4/group). For the SHIV-AE6 stock, 3/4 animals exhibited peak viral loads ranging from 6.9 to 7.3 log RNA copies/ml at 2 to 3 weeks post inoculation, but minimal CD4^+^ T cell decline ranging from 10 to 14% during acute infection (Fig 3A), and 2/4 animals were still viremic at week 10. All 4/4 animals challenged with the SHIV-AE6RM stock became infected, and 2/4 animals were viremic at week 10 (Fig 3B). For the SHIV-AE16 challenge, 2/4 animals displayed robust peak viral loads up to 7.2 log RNA copies/ml and detectable viremia at week 10, while the other two animals had low or undetectable levels of viremia (Fig 3C).

### Intrarectal titration of the SHIV-AE16 stock in rhesus monkeys

**Fig 4:**
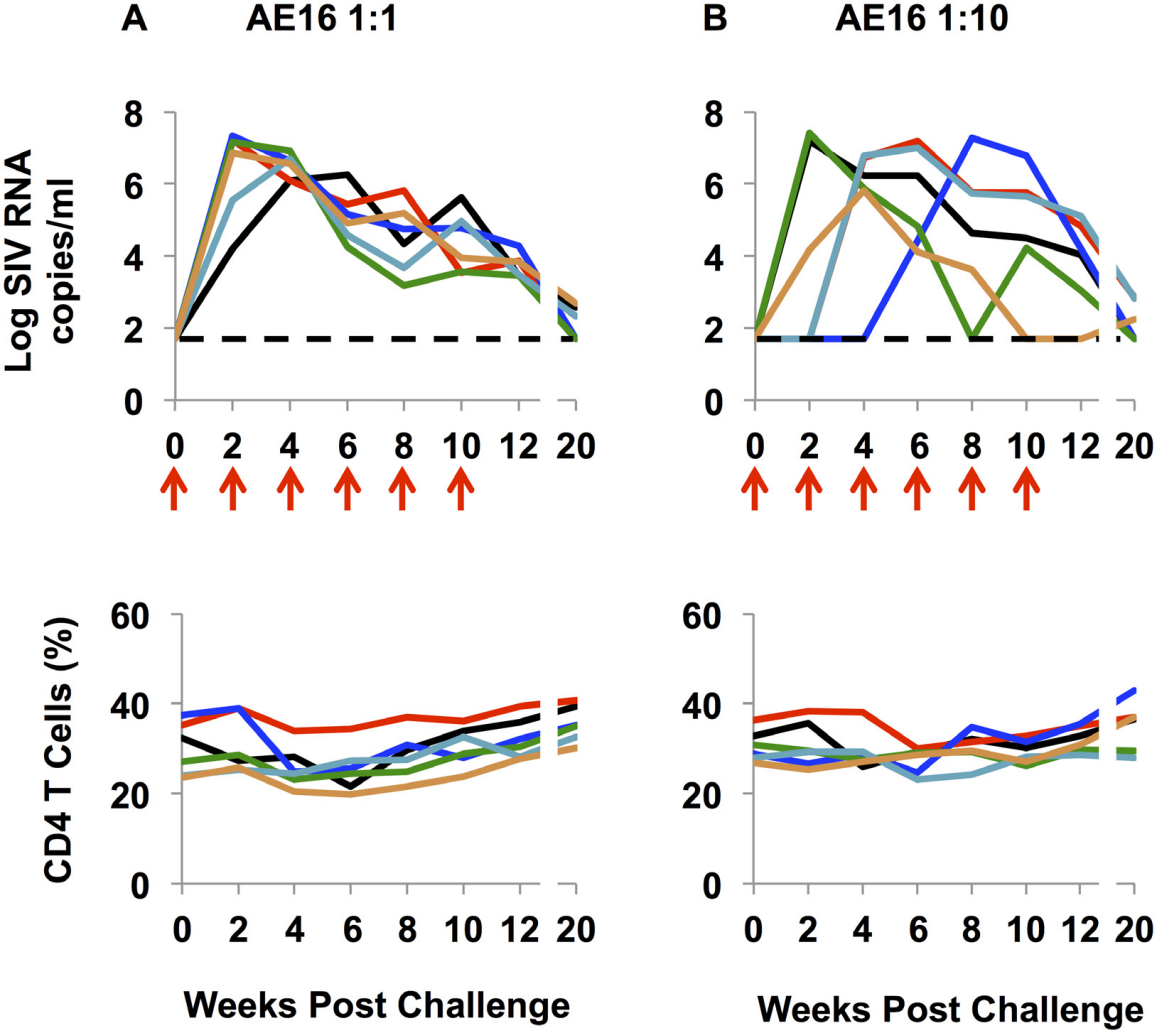
Intrarectal titration of the SHIV-AE16 stock in rhesus monkeys (1:1 and 1:10 dilution). Twelve animals were challenged six times (red arrows) with two week intervals through week 10 with 1ml of (A) 1:1 diluted or (B) 1:10 diluted SHIV-AE16 stock by the i.r. route. The upper panel shows plasma viral loads, and the lower panel shows the percentage of CD4^+^ T cells in peripheral blood. The dotted line reflected the limit of detection of the assay (50 RNA copies/ml).

SHIV-AE16 contained V2 sequences that match target epitopes of V2-specific mAbs isolated from the RV144 study [14,18]. Thus, SHIV-AE16 was chosen for further study. We conducted an *in vivo* titration experiment to determine the infectivity of SHIV-AE16 in a larger group of animals, as well as its infectivity following repetitive lower dose inoculations. Twelve adult rhesus monkeys received six repetitive i.r. challenges with either a 1:1 dilution (n = 6) or a 1:10 dilution (n = 6) of the SHIV-AE16 stock. 6/6 animals that received the 1:1 dilution were productively infected after the first challenge. These data suggest that this dose may be useful for single, high-dose challenge studies. Peak viral loads ranged between 6.1 to 7.3 log RNA copies/ml by week 2–6 and resulted in modest 6–14% declines in CD4^+^ T cell levels during acute infection (Fig 4A). Viral loads declined but were still detectable in 6/6 animals at week 10 and in 3/6 animals by week 20. In the 1:10 dilution group, 3/6 animals became infected after the first challenge, while the remaining three animals became infected after the second or third challenge (Fig 4B). Animals established peak viral loads ranging from 5.8 to 7.4 log RNA copies/ml, and viral loads were detectable in 3/6 animals by week 20. These data suggest that the 1:10 dilution may be useful for repetitive, low-dose challenge studies.

**Fig 5:**
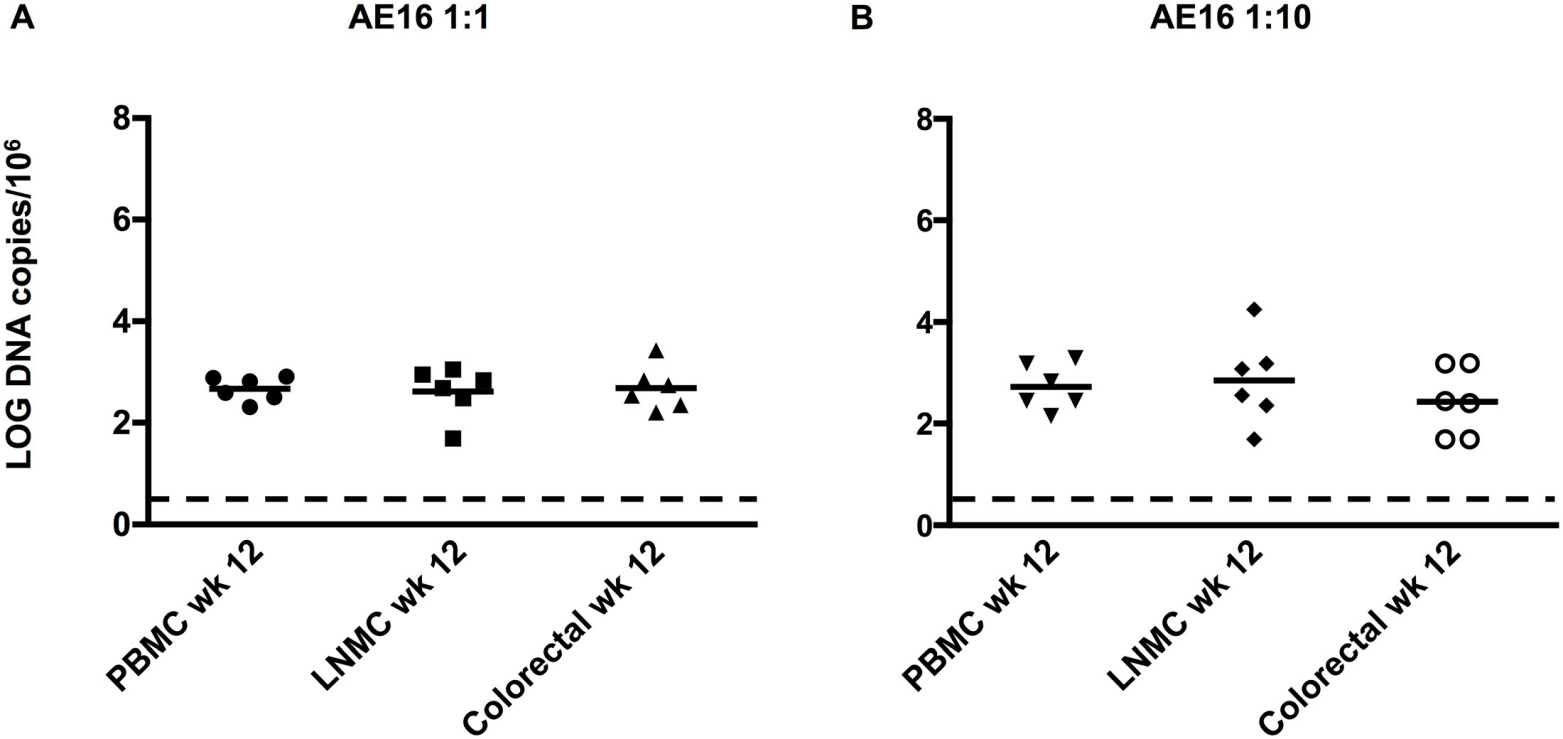
Log viral DNA copies/10^6^ cells from week 12 PBMC, LMNC, and colorectal samples obtained from rhesus monkeys infected with SHIV-AE16 stock at (A) 1:1 and (B) 1:10 dilution. The dotted line depicts the limit of detection of the assay (3 copies/10^6^ cells).

In addition to viral RNA, we also confirmed the presence of viral DNA in peripheral blood mononuclear cells (PBMC), lymph node mononuclear cells (LNMC), and colorectal cells from these animals at week 12 (Fig 5), indicating viral infection in tissues. Taken together, these data show that the 1:1 and 1:10 dilutions of SHIV-AE16 stock led to productive infection in rhesus monkeys.

## Discussion

The development of CRF01_AE SHIV challenge stocks has to date remained elusive. This has been an unmet need for the HIV-1 research field, as such viruses are critical for preclinical testing of vaccines, antibodies, and other interventions aimed at preventing CRF01_AE HIV-1 infection. In this manuscript, we describe the generation of mucosally transmissible CRF01_AE SHIV challenge stocks. These challenge stocks will likely prove useful for preclinical testing of vaccines and mAbs related to the RV144 vaccine study, which showed a modest level of protection in Thailand [6].

Our goal was to generate mucosally transmissible CRF01_AE SHIV challenge stocks expressing early HIV-1 *env* CRF01_AE sequences. SHIV-AE6, SHIV-AE6RM, and SHIV-AE16 encoded *env* sequences that closely matched the original sequences (>99%) from early HIV-1 CRF01_AE strains from Thailand. The SHIVs were highly infectious by the i.r. route in rhesus monkeys (Table 5), displayed robust acute viral replication, modest CD4^+^ T cell declines, and chronic viremia in 17/24 (71%) animals at week 10 and in 6/12 (50%) animals at week 20 (Figs 3 and 4).

**Table 5:** Summary of in vivo infectivity of SHIVs -AE6, -AE6RM, and -AE16 stocks in rhesus monkeys.

Route	Dilution	AE6	AE6RM	AE16
i.r.	None	3/4 (75)^a^	4/4 (100)^a^	2/4 (50)^a^
	1:1			6/6 (100)^a^
	1:10			3/6 (50)-1^b^
				5/6 (83)-2
				6/6 (100)-3

^a^The results after the first challenge.

^b^The results after the first, second, and third challenges. Infectivity rate values are shown in percentages (%).

Previous research has shown that *in vivo* passaged SHIVs accumulate mutations in HIV-1 *env* that subsequently increase the pathogenicity of passaged viruses [14,19]. In an effort to prevent mutations and divergence from the parental HIV-1 *env* sequences, we generated our challenge stocks without *in vivo* passaging. Despite these attempts, SHIV-AE6RM developed a single D168H mutation in the V2 loop in 20/31 amplicons (Fig 1). Mutations in the V2 loop are common in the generation of SHIVs [3], and other studies have suggested that laboratory adapted HIV-1 strains acquire positively charged amino acid mutations in variable loops that confer increased sensitivity to sCD4 (Table 4) [20]. Further studies will be needed to determine if the D168H mutation is critical for conferring adaptation in rhesus PBMC.

It is desirable for a SHIV challenge stock to be titrated for both single, high-dose challenges as well as repetitive, low-dose challenges. Vaccine studies in rhesus monkeys are often assessed by repetitive, low-dose challenges, whereas passive protection studies with monoclonal antibodies typically utilize single, high-dose challenges [21]. To develop a CRF01_AE SHIV stock that would be broadly useful, we titrated the SHIV-AE16 challenge stock *in vivo* (Fig 4). These data suggest the potential utility of SHIV-AE16 at 1:1 dilution for single, high-dose challenges and at 1:10 dilution for repetitive, low-dose challenges. In particular, this CRF01_AE SHIV challenge stock may prove useful for preclinical evaluations of vaccine regimens or V2-specific mAbs [7] related to the RV144 study to evaluate proposed mechanistic immune correlates of protection.

The infectivity and pathogenicity of our CRF01_AE SHIVs appear lower than our previously published clade B and C SHIVs [14,22]. These CRF01_AE SHIV stocks will therefore likely prove useful for challenge studies in which protection from acquisition of infection is the endpoint, but for studies of chronic virus pathogenesis, in vivo adapted virus stocks will likely be required. We note that there was higher infectivity of the SHIV-AE16 challenge stock at 1:1 dilution (6/6 animals; Fig 4A) as compared with undiluted virus (2/4 animals; Fig 3C), but the implications of this observation remain unclear given the small numbers of animals utilized in the initial study.

In summary, we report the generation of novel CRF01_AE SHIV challenge stocks. These viruses did not require *in vivo* passaging and were titrated for use in rhesus monkeys. These CRF01_AE SHIV challenge stocks should prove useful to test vaccines, antibodies, and other interventions targeted to CRF01_AE HIV-1.
